# Urban heat transitions policy; Zooming in on Dutch natural gas-free neighbourhoods

**DOI:** 10.1016/j.heliyon.2024.e37932

**Published:** 2024-09-19

**Authors:** Sanne Akerboom

**Affiliations:** Copernicus Institute of Sustainable Development, Faculty of Geo Sciences, Utrecht University, Princetonlaan 8a, 3584, CB, Utrecht, the Netherlands

**Keywords:** Energy transition, Heating transition, Policy studies, Policy interventions points, Transition typology

## Abstract

Heating transitions, where residential and industrial heating demand transitions away from fossil fuels, are an essential part of sustainability transitions. Thus far, the focus on heating transitions is neglected in policy studies, even though policy interventions are not only understood as essential, but also difficult to implement. In this article we seek to understand how policy can support the heating transition in countries with high distribution of natural gas in household consumption. By aligning transition typologies, consisting of a discussion of transition paths and innovation frames, with policy intervention points to existing literature on policy studies of heating transitions, we identify lessons learned and factors necessary for rapid heating transitions. This allows us to understand which type of transition is best stimulated through which intervention point. We then applied this framework to the Dutch natural gas phase out package, to test and validate the framework, as well as identify policy gaps and failures for the Dutch context. Here we were able to highlight important policy gaps and identified potential failures for policy effectiveness. This framework may be used in future studies and by policymakers to understand policy gaps and potential failures, supporting the (re)design of policy packages.

## Highlights

• This article presents a combined framework of transition typologies and policy interventions.
• We combined this framework with factors enabling rapid heat transitions from previous studies.
• And present a policy framework tailor-made to urban heat transitions.
• We applied this framework to the Dutch policy package and identified policy gaps.
• The framework enables future research and policymaking.

## List of abbreviations

Billion cubic metres: bcm
District heating: DH
District heating and cooling: DHC
Greenhouse gasses: GHG
Key success factors: KSF
Megaton: Mt
Nationally Determined Contributions: NDC
Petajoule: PJ

## 1 Introduction

The heating transition, where residential and industrial heating demand transitions away from fossil fuels, is an essential part of sustainability transitions. Globally, operational energy demand in buildings (including space heating and cooling, water heating, lightning and cooking) has grown to around 135 EJ, which is an increase of 4 % from 2020, whereas CO_2_ emissions from building operations have increased to an all-time high of around 10 GtCO_2_, a 5 % increase from 2020 [1]. Heating is responsible for about 50 % of total final energy consumption. Of the total demand, only a minor share of 11 % is met with renewable energy sources [2]. According to the International Energy Agency, decarbonizing heating globally is essential to meet the Net Zero Scenario, in line with the limitation of global temperature rise to 1.5 Degrees Celsius [3]. For instance, At the European Union level, 75 % of heating and cooling is still met with fossil fuels [4]. The share of fossil fuel use in the buildings sector must decline by 96 % in 2050 to align with the Net Zero Scenario. The consumption of natural gas for space heating must therefore drop to 0.5 % in 2050 [3].

Pressured by the ambition to become the first climate-neutral continent by 2050, the EU has zoomed in on decarbonising residential heat and cooling and proposes ‘a renovation wave’ [4,5]. To achieve the mid-term target of greenhouse gas emission reduction of 55 % by 2030 relative to 1990, emissions from buildings should be reduced by 60 % through promoting energy efficiency and consumption of renewable energy sources. A substantial legislative package is underway to achieve this, and the following three directives will be updated to reflect the 2030 target: Energy performance of buildings, Energy efficiency and Renewable energy sources [[6], [7], [8], [9], [10], [11]]. The Emission Trading Directive will moreover be updated to include emissions from buildings [12]. To support citizens and achieve a just transition, the Social Climate Fund will support vulnerable households to invest in renovations and sustainable heating systems. At the same time, member states are formulating individual targets and policy strategies to decarbonize heat.

Thus far, the focus on heating transitions is neglected in policy studies [13], even though policy interventions are not only understood as essential, but also difficult to implement [14]. Existing studies focusing on policy and governance often explore contexts within which low-carbon heating infrastructure is more feasible due to existing characteristics. This includes Scandinavian countries, but on the challenges of countries with high distribution of natural gas for instance, including the United Kingdom and the Netherlands, there is considerably less focus [[14], [15], [16]].

There is therefore a knowledge gap as to what policy facilitates or even accelerates heating transitions. In this article we seek to understand how policy can support the heating transition in countries with high distribution of natural gas in household consumption, thus posing a considerable challenge to realize a swift and large-scale transition to low-carbon heating systems. As such, we study this transition from a policy perspective and investigate the Dutch urban heating transition policy as a case-study. This article seeks to make a two-fold contribution. First, a theoretical contribution, by formulating a novel policy assessment framework specific to the urban heat transition. This framework is based on literature on transitions typology, policy interventions and previous studies on heating transition policies. This framework is then applied to the Netherlands, to evaluate their emerging policy framework to decarbonize residential heating consumption.

First, we briefly explain the concept and different typologies of transitions (2.1) and the types of policy interventions that enable transitions (2.2). Secondly, we focus on previous studies focusing on policy for heating transitions, which identify success factors (2.3) and identify typologies of and within ongoing heat transitions (2.4). To facilitate and achieve success factors of urban heating transitions, we then combine the success factors with the typologies and subsequently the policy intervention points, to identify which interventions can achieve the success factors (2.4). In section 3 we then analyse the Dutch urban heating transition policy, partly based on the policy mixes for sustainability transitions framework [3], after which we apply the combined framework to assess this policy [4] and discuss our findings [5].

## 2 Urban heat transitions

### 2.1 Definition and typology of transitions

Sustainability transitions can be defined as a radical transformation towards a sustainable society, responding to several persistent problems confronting contemporary modern societies [17]. Transition studies originate from innovation studies and has been dominated by scholarship on the Multi-Level Perspective (MLP) [18,19], Technological Innovation Systems [20,21], Strategic Niche Management [22,23] and Transition Management [24,25]. Scholars have tried to capture different types of transition pathways and innovation frames. Schot and Geels [23] for instance have defined six transition paths, whereas Schot and Steinmueller [26] focus on three dominant frames for innovation policy.

Geels and Schot [23] define six transition paths, attempting to overcome criticisms on the MLP, including the empirical legitimacy, neglect of agency and focus on technological niches as the principal instigator of regime change. Rather, they argue, regime change can come about due to a variety of different pressures on the regime, leading to different transition paths. Based on this, Schot and Geels present six transition paths. The *reproduction process* (PO) depicts a stable regime, where technology reproduces. In the *transformation path* (P1) there is moderate landscape pressure, for instance from outsiders such as interests’ groups or social movements, but at a moment where niche-innovations have not yet sufficiently developed. *De- or re-alignment paths* (P2) come about when the regime faces significant pressure. If regime actors lose faith, the regime may erode or de-align. Even if multiple niche-innovations arise, often, one becomes dominant and re-alignment with a new regime takes shape. When a niche-innovation has developed sufficiently, it can replace the existing regime. In this case, there is a *technological substitution path* (P3). When niche-innovations are adopted in the regime to solve local problems and trigger further adjustments in the basic architecture of the regime there is a *reconfiguration path* (P4). When several of these paths take place at the same time, especially considering disruptive change, there is a *sequence of transition pathways* (P5).

Innovation frames on the other hand depict “interpretations of experience, ordering of present circumstances and imaginations of future potentialities that create the foundations for policy analysis and action and shape expectations concerning potentials and opportunities” [26]. By focusing on three frames, Schot and Steinmueller attempted to articulate frames for science, technology and innovation policy that emphasizes socio-technical system change. The first frame concerns *innovation for growth (TF1)*, focusing on technology to enable prosperity but also mass production and consumption. The second frame, *systems of innovation (TF2),* aims to address (negative) consequences of modern economic growth for individual states. A third frame – *transformative change (TF3)* – is proposed in response to the need to meet social goals such as ending poverty, reducing inequality, promoting inclusive and sustainable consumption and production systems, also known as the UN Sustainable Development Goals.

### 2.2 Policy interventions for transitions and policy mixes for sustainability transitions

Policy studies focus on the potential of different policy mixes and interventions to instigate, accelerate, and achieve sustainability transitions. Within transition studies, two type of policy evaluation frames have emerged: policy instruments for sustainability transitions and policy interventions points.

The study of *policy mixes for sustainability transitions* has flourished: literature on policy mixes for sustainability transitions has focused on ‘ideal’ packages of policy instruments, whether such packages are successful in meeting the policy goals and how policy mixes can be designed [27]. Rogge and Reichardt put forward a framework for the evaluation of the policy mix, on the basis of three buildings blocks: 1) policy strategy and goals, in order to describe the objectives of the policy and which instruments are envisaged in order to meet these objectives; 2) policy processes, to describe the background and context and how the policy strategy and objectives came about and 3) characteristics of this strategy and instruments, evaluated on the basis of four elements: consistency, coherence, credibility and comprehensiveness [27].

Kanger et al. [28] in contrast identify *policy intervention points*: “particular areas in the socio-technical system or its environment where the application of appropriate policy instruments would likely facilitate transformative change in the system's directionality.” Policy intervention points differ from policy strategies as an intervention point clearly focuses on what to target and why targeting would be a good idea, whereas a strategy puts emphasis on measurable targets and concrete plans. Following this definition, Kanger et al. identify six intervention points [28]. The first, *stimulate different niches (IP1)*, aims to sustain variety of (technological) solutions. Niches are developed differently, some need time to mature. Opting into a single option, effectively eliminates new niches. Rather, it is better to present a pool of alternative solutions for challenging and transforming the incumbent regime [23]. Measures to stimulate niches are R&D funding schemes, public procurement, or regulatory conditions. Secondly, it is important to *accelerate niches (IP2)*. This concerns both scaling-up and alignment of niches. Means to accelerate niches are market-based policy instruments, promotion of entrepreneurship or provision of venture capital. To allow niches to break-through, a third intervention point is to *destabilize the regime (IP3)*. Measures include taxes, banning or phase out of specific technologies or removing subsidies. The fourth intervention point is to *address the broader repercussions of regime destabilization (IP4)*. Different impact may occur, such as of physical infrastructure, but also culturally or financially. Measures to address effects of regime destabilization include payments to industry for phasing out fossil-fuels, financial and educational support for unemployment and skill mismatch or regional diversification of industrial activities. Different regimes may influence each other. Measure should therefore be introduced to *coordinate multi-regime interaction (IP5)*. There is a need to move beyond the current landscape and niches, and therefore the sixth and last intervention point is meant to *tilt the landscape (IP6)*. Measures here include participation and international negotiations and binding agreements. Kanger et al. combined the six intervention points with the typology of transitions from Schot and Geels [23], identifying key intervention points to achieve the transition pathway [28]. An overview of the policy intervention points and transition paths is provided in Table 1 below.

**Table 1:** Policy intervention points and transition paths (Kanger et al., 2020).

Intervention point	Underlying aim	Examples	Key intervention points for transition path/frame
Stimulate different niches	To guarantee the presence of various alternatives for systems change	Subsidies and regulatory shielding for niches in various systems	De-alignment and re-alignment
Accelerate the niches	To scale up single niches and to align different niches to each other	Providing support for entering the markets and/or creating links between various niches	Transformation, de-alignment and re-alignment
Destabilize the regimes	To weaken the position of incumbent regime actors hindering the transition	Removal of various forms of regulatory and financial protection favouring incumbents	Reconfiguration
Address the broader repercussions of regime destabilization	To anticipate and manage the broader societal impacts resulting from systems change	Provision of financial and educational support for structural unemployment and skill mismatch	Substitution
Provide coordination to multi-regime interaction	To ensure that the input-output relations between the regimes would be complementary	Devising mutually supporting interventions in multiple systems	Reproduction
Tilt the landscape	To alter the broader framework conditions enabling change in the directionality of locally bounded socio-technical systems	Participation in international negotiations to arrive at collectively binding agreements	Reproduction

### 2.3 Heating transitions and policy lessons learned

Recently more research has been dedicated towards heating transitions, e.g., the decarbonisation of heating demand in residential and industrial end-use, as an essential part of the energy transition. Sovacool and Martiskainen [13] describe that the heating sector, especially in residential demand, is one of the hardest sectors to decarbonize, and that they are associated with long-lived infrastructure, costly alternatives and patterns of incumbency and path dependence.

Many studies focus on the range of technological options for decarbonizing heat. In some cases, end-users are connected to district heating grids (DH), which uses fossil heating sources including natural gas, and DH needs to be decarbonized [29,30]. This can be achieved through so-called ‘fourth generation DH’, which aims at improving energy efficiency through the reduction of heat losses of the network and the utilization of renewable energy sources [31]. Some studies even focus on fifth generation DH which includes (sustainable) cooling [32]. For countries like Sweden and Denmark decarbonizing DH is the biggest challenge, as they have opted for DH decades ago, but still make use of fossil fuels [30,[33], [34]]. In other cases, residential end-users rely directly on natural gas for their heating demand, and heating transitions can entail a multitude of technological solutions, including new DH grids or individual solutions such as heat pumps. Heating transitions also include options such as retrofitting and refurbishments of buildings or standards for energy efficiency for new buildings [3,34]. For countries like the Netherlands, phasing out natural gas and opting into new technologies is key as they switched from coal to natural gas in the 1960s and a large share of residential heating demand is currently met with natural gas [34,35].

Perhaps the most substantial challenge lies in the transition away from natural gas. Decarbonizing DH has less impact on end-users as the consumption nor the technology used needs to change. Transitioning away from natural gas for direct residential consumption entails technological and behavioural changes within the home, including structural changes of infrastructure and cooking and heating utilities. This can also bring forth considerable costs [36,37]. Of course, there are less challenges in case renewable gas can be introduced, but there are some limitations to this possibility [38].

Policy studies on realizing heating transitions are coming up [[39], [40], [41], [42]]. A prime example of this concerns the study of Sovacool & Martiskainen [13], in which the governance of rapid transitions in four case studies is evaluated. In this study, Sovacool and Martiskainen find that the proper design of governance is pivotal to the success of these challenging transitions. From 4 case studies, they have identified 24 dimensions within 6 categories that aid rapid urban transitions. These six categories concern: 1) equity and co-benefits, 2) inclusivity and local involvement, 3) information, demonstration, and innovation, 4) ownership and accountability, 5) organizational multiplicity and 6) experimentation and flexibility.

Moreover, a 2016 study by the Joint Research Council investigated eight district heating and cooling (DHC) case-studies in seven European countries, to identify key success factors (KSF) for the development of efficient DHC systems [43]. Based on 9 KSF's, the researchers unlock four potential implications on policy guidelines. The nine key success factors concern: 1) adequate national policy and regulatory environment, 2) financial support, 3) local policy and coherence with urban planning, 4) alignment of interests and cooperation, 5) availability and relevance of local resources, 6) comprehensive project development, 7) price competitiveness, 8) flexibility and 9) combining technical and non-technical innovation. This leads to four policy implications. First is to support the in-depth design and comparison of optimal new local systems. Given the variety of technological options, and the characteristics of building necessitate that all options should be considered. Second, regulatory patterns must be revisited. DHC is usually under control of local authorities, supply schemes and key features can strongly differ, and this does not suit classical centralised regulation. In experienced countries like Denmark and Sweden, there is often a combination of centralised regulation, which contains generally applicable rules such as consumer protection, and local control, allowing for strong consumer involvement. Thirdly, system integration of cooling, heating and power is recommended. Lastly, DHC should be part of larger multi-energy systems and be included in smart grid and innovation programmes.

There is some overlap between the 6 dimensions and 9 KSF: both innovation and involvement of consumers and cooperation between (local) parties are deemed important and it is recognised that heat transitions cannot be successfully established without financial support, preferably a combination of direct and indirect incentives. In other points, these frameworks are complementary: Galindo Fernández et al. [43] focus on the role of regulation, and a holistic approach of balancing alternative heat systems, including in relation to prices and tariffs. Sovacool and Martiskainen further develop the dimensions on experimentation, demonstration, and innovation [13]. Together, these two studies highlight the best-available knowledge on collective heat systems and the transition towards a sustainable urban environment.

Below we use the overview presented by Sovacool and Martiskainen [13], and supplement the elements with two new categories, as presented by Galindo Fernández et al. (43: holistic approach and adequate national policy and regulatory environment and thirteen new dimensions within these dimensions. Furthermore, we add three new dimensions to existing categories: 1) to focus also specifically on prices in the category of inclusivity and local involvement; 2) to combine non-technical and technical innovation in the information, demonstration, and innovation category and 3) to align interests and comprehensive project development to the category of organizational multiplicity. Therefore, the combined factors represent eight categories and 40 dimensions, which are presented in Table 2 below.

**Table 2:** Factors for rapid heating transitions.

Category	Dimension
Holistic approach (NEW)^a^	1) Options of DHC balanced against alternatives, including environmental and social benefits 2) Availability and relevance of local resources 3) Methodology at the national level 4) Price competitiveness, throughout lifespan 5) Flexible heat and cold production through diversified and complementary optimisation
Adequate national policy and regulatory environment (NEW)	6) Balance of national and local regulation 7) Connection of heat regulation and urban planning 8) Set of policy tools present 9) Presence of financial incentives: taxes and support schemes 10) Ambitious sustainability targets 11) Study relevance of (temporary) mandatory connections at a local level 12) Transparency of prices 13) Incentives for a competitive energy supply over time
Equity and co-benefits	14) Use of public funds, loans, subsidies, and taxes 15) Cost-sharing 16) National policy targets 17) Co-benefits and positive externalities
Organizational multiplicity	31) Various government actors (local, municipal, provincial, national) 32) Industry and industrial organizations 33) Civil society groups and research institutes 34) Transnational actors 35) Align interests (NEW) 36) Comprehensive project development
Inclusivity and local involvement	18) Emphasis on domestic use 19) Prioritization of rural areas 20) Involvement of users, in relation to prices NEW) 21) Incentives for local private firms and enterprises
Ownership and accountability	27) Household or community ownership 28) Improvement of standards 29) Compulsory use requirements and/or penalties for non-compliance 30) Scaling back of subsidies
Information, demonstration, and innovation	22) Dissemination of data, information, and knowledge 23) Active marketing or demonstration programs 24) Technological learning 25) Innovations in technical performance 26) Combine technical and nontechnical innovation (NEW)
Experimentation and flexibility	37) Experimentation in technical design 38) Multiple uses, applications, or configurations 39) Progression of scalable targets 40) Adaptability in transition management

a The mention NEW is relative to the table presented in Sovacool and Martiskainen (2020) and stems from the research of Galindo Fernández et al., 2016.

### 2.4 Typology of urban heat transitions

Both the transition typology pathways and innovation frames are relevant to the urban heat transition. Depending on the context and circumstances of countries, we can distinguish several transition paths within heating transitions. The urban heat transition has far-reaching consequences. It requires both technical innovation and behavioural change. The urban heat transition can therefore be identified as *transformative change*. Schot and Steinmueller argue that this model of innovation is inherently experimental, as possible pathways are unknown. Possible pathways and the required knowledge base can moreover only be found through collaboration, participation, and inclusiveness, by means of deliberation, societal experimentation and learning and public debate, to articulate new needs and demands, with an accumulation of experience by a variety of actors [26].

This has implications for policy. Possible failures include the failure to let social choices prevail in new pathways and to only consider options and boundaries set by incumbents. There is the possibility of failure to coordinate diverse policies, limiting the options of a holistic approach of the transformative change. Societal experimentation and learning moreover requires reflexivity. The failure to reflect on learning can limit the ability to look beyond deeply embedded routines. Therefore, policy should not only move beyond incumbency, but contribute to a process of destabilization of existing socio-technical systems and navigate existing policies and create new policies [26]. There is a strong connection between these identified potential policy failures and the recommendations of Sovacool & Martiskainen [13] and Galindo Fernández [43], as they also recommend keeping options open through a holistic approach, move beyond incumbency and deeply embedded routines and to allow for social experimentation. Several policy interventions points align with this, including stimulating different niches, destabilizing the regime, and coordinating multi-regime interaction.

To study policy interventions with the potential to realize and accelerate urban heating transitions we combine the factors for rapid heating transitions with transition typologies and subsequently policy interventions points, to understand which interventions can achieve the success factors. An overview of the combined framework is provided below in Table 3.

**Table 3:** Combined framework: policy interventions points for successful urban heating transitions.

Category	Transition typology/frame	Policy intervention point
Holistic approach (NEW)∗	De/re-alignment (P2) Transformative change (TF3)	Stimulate different niches (IP1) Accelerate niches (IP2)
Adequate national policy and regulatory environment (NEW)	Reproduction (P0) De/re-alignment (P2) Reconfiguration (P4) Transformative change (TF3)	Accelerate niches (IP2) Destabilize the regime (IP3) Coordination multi-regime interaction (IP5)
Equity and co-benefits	Substitution (P3) Reconfiguration (P4)	Destabilize the regime (IP3) Address the broader repercussions (IP4)
Inclusivity and local involvement	Substitution (P3) Reconfiguration (P4)	Destabilize the regime (IP3) Address the broader repercussions (IP4)
Information, demonstration, and innovation	De/re-alignment (P2)	Stimulate different niches (IP1)
Ownership and accountability	De/re-alignment (P2) Substitution (P3) Reconfiguration (P4) Transformative change (TF3)	Accelerate niches (IP2) Destabilize the regime (IP3) Address the broader repercussions (IP4)
Organizational multiplicity	Reproduction (P0) De/re-alignment (P2) Transformative change (TF3)	Stimulate different niches (IP1) Accelerate niches (IP2) Tilt the landscape (IP6)
Experimentation and flexibility	De/re-alignment (P2) Transformative change (TF3)	Stimulate different niches (IP1) Accelerate niches (IP2)

With this in mind, we will now explain the Dutch policy framework on natural gas phase out in the build environment as a case study (section 3, 4).

## 3 Case study: Dutch urban heating transition

The Netherlands aims to make its built environment more sustainable. The diffusion of natural gas in the Netherlands is widespread, as collective grids were realized in the 1950–1960s after the discovery of natural gas resources in the northern province of Groningen. The transition away from natural gas was pressured by two developments: the need to mitigate global climate change and earthquakes in the northern province Groningen, where most of the domestic gas explorations take place, and since the winter of 2021/2022 the dramatic increase of natural gas prices presents a third pressure to accelerate the Dutch heating transition.

Therefore, the Netherlands has opted to phase out natural gas in the built environment. In this paragraph we apply the framework of policy mixes for sustainability transitions [27] to analyse the policy strategy, goals, and instruments (3.1) and how the policy processes came about (3.2). These two building blocks are descriptive in nature. The framework consists of a third building block, concerning the characteristics of the strategy and instruments. Instead of this step, we will apply the new conceptual framework as presented in section 2 (section 4).

### 3.1 Policy strategy and goals

Over recent years, the Netherlands has continuously created more ambitious emissions reduction targets. A new Dutch Climate Act entered into force in 2019, stipulating a greenhouse gas (GHG) emission reduction target of net-zero by 2050, relative to 1990. This target will be updated to reflect the 2030 and 2050 targets from the EU Climate Act (e.g., 55 % and net-zero, 44). Measures to realize this target have been agreed upon in a public-private agreement, the so-called Dutch Climate Agreement [45]. Five sectors, electricity production, agriculture, mobility, industry and built environment were each awarded a specific reduction target. Within the built environment a reduction of 3.4 Megaton (Mt) CO_2_ emissions is expected by 2030 as a first step in this sector. However, this was recently increased to 7 Mt due to the new EU and Dutch targets. As a first step, 1.5 of 8 million houses must become sustainable by 2030, and by 2050, all buildings and houses must be natural gas free [45]. There are various technological solutions to achieve this [46].

Within the built environment, ambitions will be realized through the following coordinated approach: new buildings and houses should no longer be connected to the natural gas infrastructure, but should rather opt for an alternative heating system, such as district heating or heat pumps. The existing built environment will gradually become more sustainable [39]. District heating will be stimulated through a new Collective Heating Act.

Most emphasizes in policy is placed on collective heat systems. Although individual homeowners can decarbonize their heat consumption by opting for a heat pump for instance, most policy instruments aim at offering support for collective systems. Below we will mention per instrument how it is aimed at collective or individual options.

Below the policy strategy and instruments will be described into more detail, based on the policy mixes for sustainability transitions framework. First, the policy instruments for new buildings will be discussed in 3.1.1, but this will not be used further. Secondly, the policy instruments to phase out natural gas from existing buildings and houses will be discussed in 3.1.2. As this presents the greatest challenge in the Netherlands, we will focus on this.

#### 3.1.1 New buildings and houses and Dutch policy

A first step towards natural gas free neighbourhoods was made when the Energy Transition Acceleration Act (in Dutch: Wet versnelling energietransitie) entered into force on July 1, 2018. This Act determined that new buildings and houses cannot be connected to the natural gas grid. With this Act, the obligation for gas distribution system operators (DSOs) to connect new consumers to gas grids was cancelled. Before July 2018, DSOs had the obligation to connect anyone to the gas infrastructure upon request. Instead, the Board of Mayor and Aldermen at the municipal can now determine for which new areas a collective heating infrastructure will be realized or opt for individual solutions such as heat pumps. However, in some cases the municipality can determine that a connection to the gas grid is imperative, for instance after a societal cost-benefit analysis, and opt for an exception clause that allows some new neighbourhoods to be connected to the gas grid.

Furthermore, the government, together with consumer organizations, builders, grid operators and energy companies, closed an agreement New Built Natural Gas Free (in Dutch: Akkoord Nieuwbouw Aardgasvrij) in which they agreed to deliver 75 % of all new buildings and houses natural gas free between July 1, 2018 and late 2021. It was also agreed that existing buildings and houses that can be made natural gas free relatively easy should receive financial support. Mortgage lenders for instance can increase the mortgage amount specifically for this cause. Other options, such as loans, would be investigated.

Since July 2018, 74 municipalities have opted for the exception clause [47]. In Amsterdam for instance, 22 % of all new buildings and houses are still connected to the gas infrastructure. There are large provincial differences, in Groningen only 10 % of all new buildings and houses are connected to the gas infrastructure, this concerns 3 % in Flevoland but 30 % in Limburg [48]. Whether the goal of 75 % will be met remains to be seen. Depending on the local characteristics, the municipality can opt for individual or collective systems for new neighbourhoods. If necessary, developers could make use of subsidies for collective heating systems. For individual solutions, such as heat pumps, these are priced into the house, or can be purchased or rented by the future homeowner. In case the homeowner purchases the heat pump, there might be subsidies available, see under ‘Financial instruments’ below.

#### 3.1.2 Existing buildings and houses and Dutch policy

In the Dutch Climate Agreement, parties agreed to steps for the period up to 2030, to realize the target to make 1.5 million existing households more sustainable, and an extensive package of policy instruments was introduced. This package consists of plan- and decision-making and financial instruments, the intention to draft a new Heating Act to replace the current Act and a learning program. Integral part the policy mix is the focus on the social aspect of the urban heating transition. Therefore, decision-making will be characterised by co-creation, and plans and decision regarding alternative heating systems will be made together with residents. Below I will provide a comprehensive overview of the policy framework. This framework is made up by a Heat Vision [59], Energy Report [59], two Coalition Agreements [44,51] and Dutch Climate Agreement [45]. New or adaptations to existing policy instruments stem mostly from the latter two policy documents. I have selected the most important instruments at the national level in several categories: planning and decision-making, learning program, financial instruments, regulation, and technical standards. There may be additional instruments at the local level, but these fall outside the scope of this study.

##### 3.1.2.1 Planning and decision-making instruments

The main governmental actor in the Dutch heating transition is the municipality. The municipality brings together parties to determine technological alternatives to natural gas and timeframes etc. (Herreras Martinez et al., 2022). The planning and decision-making process for alternative heating systems concerns a programmatic approach. A distinction is made between projects for large-scale apartment complexes and neighbourhoods consisting of houses. For large-scale apartment complexes with many renters, so-called starter motor projects will be initiated. This will allow for large-scale plans to make apartment complexes sustainable. Renters should be presented with multiple options and a Renovation Accelerator Team will develop standards offerings.

Choices for alternative heating for neighbourhoods mainly consisting of houses are made based on so-called ‘neighborhood plans’, drawn up by the municipality in co-creation with residents and relevant stakeholders. Ultimately, all neighborhood plans are combined in a municipal ‘heating transition vision’, which is the starting point for realizing the plans. How this participation will be organised, can be determined by municipalities. To help guide the process, several guidelines and toolboxes will be drafted.

##### 3.1.2.2 Pilot projects and learning program

Lastly, a learning program was set up. At first, 27 pilot projects were appointed, to start with the neighborhood plans, focus on co-creation and participation and to draw up alternative heating system plans. A national Program Natural Gas Free Neighbourhoods (Dutch: Programma Aardgasvrije Wijken) has been set up to evaluate and learn from these experiences and transpose these in regulation and policy support. In later phases more pilots will be appointed and ultimately, there will be 100 pilots. Moreover, a Centre for Heating Expertise will bring together the pilots, arrangements and standards and offer technological and business support to project developers.

##### 3.1.2.3 Financial instruments

There are several financial incentives. Firstly, sustainable heating sources will be (partly) subsidized by the Dutch government under the so-called SDE++ scheme (in Dutch: Stimulating Sustainable Energy, see also 52). Energy project developers, such as wind and solar farms, but also of renewable gasses and heat sources, are eligible to receive this subsidy. This subsidy is therefore open to projects both in new and existing buildings and houses. Another important financial incentive concerns the agreement to increase gas consumption taxes. This way, the consumption of natural gas becomes more expensive, and alternative heating sources more attractive. The tax will increase gradually over the next decade. This plan however is derailed due to the extreme increase in natural gas prices, resulting in a tax reductions and price ceilings over the winters of 2021–2022 and 2022–2023.

##### 3.1.2.4 Regulation

Moreover, the legislator committed to propose a new Collective Heating Act, to replace the current Heating Act. The current Heating Act entered into force in 2013, when the heating transition did not play a role in policy or regulation. Given the multiple goals of the Dutch heating transition – societal acceptance, cost reduction and sustainability – parties to the Climate Agreement felt it necessary to reconsider the regulatory framework and guarantee it would be fit-for-purpose. The newly proposed Act contains sustainability targets, tariff regulation and consumer protection rules [53]. The Proposal Act is contested by many parties and plans to discuss this proposal in Parliament are significantly delayed.

##### 3.1.2.5 Technological standards

Alternative heating systems are still quite expensive. Upscaling technologies can contribute to lowering costs. By 2030, costs should be lowered by 20–40 %. Standardized offers for alternative heating systems and energy efficiency will help municipalities and citizens choose the best alternative option for them. Sustainability targets, to be laid down in the new Collective Heating Act will ensure that greenhouse gas emissions are gradually reduced.

### 3.2 Policy process

#### 3.2.1 Background and context

The decision to phase out natural gas in the Netherlands by 2050 hinges on two considerations: 1) earthquakes in the northern province of Groningen caused by gas exploration leading to diminished societal support and 2) the sustainability transition towards a carbon-neutral society.

Pressured by public tension due to the intensified earthquakes in Groningen, the government was losing its license to operate for natural gas production in that region. Although the Dutch government decided to gradually decrease the annual production, new decisions on the gas production in the area were all annulled by the Judicial Division of the Council of State (highest administrative court) as the decisions did not take the interests of citizens in the area enough into account [54,55]. This led to an accelerated decrease of production.

Simultaneously, the Netherlands recognised that deep decarbonisation, to meet the Paris Climate Agreement target, necessitates the phase out of natural gas. In 2012, heating demand made up 55 % of the final energy consumption, circa 1200 Petajoule (PJ), of which 44 % concerns industrial demand, 29 % of households, 20 % of the utility industry and 7 % of agriculture [49]. Only 3.6 % of the heating consumption is produced sustainably. Natural gas is responsible for 92 % of all heat production and for 13 % of all CO_2_ emissions in the Netherlands in 2018 [56]. The built environment is responsible for 14 % of all CO_2_ emissions in 2018, of which 70 % is caused by households, considering both electricity and heating demand [57]. Natural gas and heating demand therefore play a vital role in Dutch decarbonisation processes.

Both the societal pressure to lower gas production in Groningen, coupled with the need to protect energy security and remain independent from other countries with respect to energy import as well as the need to decarbonize increased the awareness of the role of natural gas and the necessity to phase this out in the Netherlands. Recent geopolitical circumstances, and the subsequent soaring natural gas prices further demand a phase out of natural gas. The stark increase of prices however has also stimulated alternative solutions, and those financially capable are currently installing solar PV and heat pumps at an accelerated pace.

#### 3.2.2 Decision-making process on the Dutch heating transition

The decision-making process can be considered swift: in 2013 the focus to achieve sustainable heating was energy saving and efficiency was still lacking, this was firstly introduced in 2015, whereas by 2017 the decision was made to phase out natural gas. This is illustrated by the Energy Agreement of September 2013 [58]. At this point in time, there were no plans to phase out natural gas consumption in the built environment yet, there was only focus on energy efficiency and decentralised production of sustainable energy.

An important policy document of April 2015 first instigated the plans when the minister of Economic Affairs sent his Heating Vision to Parliament. In this Vision [49], the minister explained the role of heating in the transition towards a sustainable energy system in 2050 and the potential of sustainable heating, and possible scenarios to phase out natural gas including district heating and all electric neighbourhoods. To this end, local heating plans had to be drawn up by municipalities to stimulate the use of residual heat and to draft plans for future heating systems in cities. Thus far, no regional heating plans for industrial heat consumption have been drawn up whilst many municipalities drafted local heating plans. To stimulate the consumption of alternative heat sources, the minister proposed to evaluate the current regulation as well as financial incentives to identify and eliminate barriers. He did not mention specific targets or timelines [49].

In 2016, the government decided to make CO_2_ emission reduction focal to all energy transition policy [52], resulting in a target of 80–95 % CO_2_ emission reduction by 2050 in comparison to 1990 [50]. Low temperature heating, used to heat houses, buildings, and greenhouses, was seen as low hanging fruit to realize this target. To this end, the government decided that CO_2_ emissions in these sectors must be brought done to 0 % by 2050.

After general elections in March 2017, the newly elected government presented its plans for the next four years. In their agreement “Trust in the Future”, the new coalition decided to make 30.000 to 50.000 houses natural gas free by 2021 and to further increase this task to 200.000 natural gas free houses per year after 2021, ultimately 1.5 million households by 2030 [51]. With this decision, the plan to eliminate CO_2_ emission from the heating demand in the built environment are solidified. The strategy and policy instruments to achieve this goal have been specific in the public-private Dutch Climate Agreement [45]. In 2021, the reduction target increased significantly, resulting in a new target for the built environment and some new instruments (see section 3.1.2). The increased gas prices of 2022 further stimulate a full natural gas phase out.

These plans were elaborated upon by the new government in November 2021, who put the focus on energy savings, heat pumps and district heating. To this end, the national government introduces a long-term national insulation program for which € 3.5 billion will be allocated, with specific focus on renters in poorly insulated houses. Three technological solutions are presented: obligation to install hybrid heat pumps as of 2026 and district heating, both in combination with a subsidy for households and incorporation of green gas. However, before these plans could be implemented, new elections were held in November 2023. Although the new government has not yet published elaborate policy strategies and plans, it has been announced that the general energy transition goals will remain. However, it has been decided to at least remove the obligation to install hybrid heat pumps. Since the precise policies and setting of the new government are still in development whilst this paper is written, the exact impact is unknown.

## 4 Evaluating the Dutch policy context: transition typologies and frames, eight categories and corresponding policy interventions

**Transition typologies and frames.** Relating the Dutch urban heat transitions to the six transition paths shows that several are applicable. First, since there is significant external pressure to change, we can identify the de- or re-alignment path (P2) present. How this change comes about however, does not follow one single path. For instance, technological focus is put on DH as a viable large-scale solution, but it is understood that this cannot be the only solutions. Therefore, we cannot identify the presence of a technological substitution path (P3). Although in terms of policy strategy, district heating appears to be a viable technological alternative, it will not be sufficient to replace all heating demand currently met with natural gas. This is therefore not yet a re-alignment or substitution path, but rather a transformation path (P1) coupled with a reconfiguration path (P4), where niche-innovations have not yet sufficiently developed, and there are significant adjustments to basic architecture proposed. As such, we can identify the sequence of transition pathways (P5). Of the three innovation frames, we identify the presence of the transformative change frame (TF3). After all, the Dutch urban heat transition responds to climatic issues, social injustice (Groninger earthquakes) and rising energy poverty. It presents a substantial technological challenge, of which the outcomes are unclear and can develop along different paths.

**Holistic approach.** Since the heating transition in the Netherlands is developing along different transition pathways (P5 and TF3), several technological alternatives to natural gas are possible. These alternatives can moreover be implemented by several different parties. The technological options available are DH, heat pumps, all-electric, green gasses and potentially (green) hydrogen [46]. The available options are considered at the municipal level, when drafting the heat transition vision per neighborhood. Depending on the technological options, they may be implemented by existing heat companies (DH) or through bottom-up initiatives, such as heat cooperatives. Individuals could opt into heat pumps and other all-electric solutions, whereas for green hydrogen and green gasses new companies may arise.

How municipalities ultimately opt between the options is unclear. They may consider the technical characteristics of the buildings, which include the age, level of insulation or potential for upgrading or retrofitting the building(s), availability of local and regional heat sources – which may include water-based heat sources such as ground water of wastewater, residual heat from biomass power plants, industry, or waste incinerator plants – possibilities for connecting to existing DH and the preferences of the residents. There are no guidelines provided by the national government for making this choice, and it is unclear how price and tariffs are considered as part of the options available. Municipalities therefore chose their own decision-making criteria.

Amsterdam for instance, has drafted a list of 5 decision-making criteria guiding the decisions in the different neighborhoods: lowest societal costs; existing infrastructure; availability of local sources; sustainability and least inconvenience. To determine the lowest societal costs, Amsterdam has contracted a consultant, who developed a financial-technical analysis for this challenge and has provided a financial report per house-type [59]. Rotterdam has not formulated comparable criteria, but also focuses on lowest societal costs and availability of resources. It has however communicated a set of 6 criteria determining the planning of the heat transition: compensation of costs and capacity at the municipal level; sufficient finances available and capacity at housing associations; finances available for collective heating systems; including for individuals residents; fit-for-purpose regulatory frameworks and connection to spatial planning [60].

The lack of nationally developed guidelines or criteria for selecting decarbonizing options may result in (large) local differences. It is also yet unclear how the locally developed guidelines or criteria will affect the choices, and account for local preferences by residents. As such, there is no explicit policy that aims to stimulate different niches (IP1).

**Adequate national policy and regulatory environment.** The policy and regulatory environment for the Dutch heating transition has been presented in section 3. This shows several policy instruments, mostly related to the planning of the transition, some financial instruments, and a new regulatory Act specifically for collective heating systems, which should be implemented on the short term, although it has not yet been send to Parliament for discussions. Unlike the recommendations of Galindo Fernandez et al. [43] and Schot and Steinmuller [26], this Act presents centralized, generally applicable rules concerning the decision-making procedure, consumer protection, tariffs, and sustainability targets. It leaves no room for local regulation, other than the planning aspect. The Act is therefore not in line with several policy recommendations stemming from previous research [53].

At the same time, it does have the ambition of creating more transparent and diversified price and tariff regulation. Ultimately, per type of collective heating system, a separate tariff scheme will be drafted. Unlike centralized and collectivized systems such as electricity and natural gas, collective heating systems will have tariff schemes depending on the local system, specific to that local system. This Act is therefore explicitly focused on accelerating the collective heating systems niche (IP2). At the same time, the wider policy framework does not aim to destabilize the regime (IP3), as it does not focus on policy instruments for the phase out of natural gas, only on the stimulation of niches, which constitutes an important gap within the policy framework. It is moreover unclear how the policy framework coordinates multi-regime interaction (IP5).

**Equity and co-benefits.** A transition of this scale is likely to bring forth societal costs. It has therefore been one of the pivotal points of political discussions to keep societal costs as low as possible. The criterion of lowest societal costs has therefore been officially introduced in the Dutch Climate Agreement and is extensively used by municipalities in their heat transition visions. There is however no operationalization of what this criterion means, or how this has been achieved. Some financial instruments have been introduced, but there are still considerable costs associated to the sustainable solutions. One of the options that is under discussion is to create mandatory connections to collective heating systems, to keep costs as low as possible and create a business case for heating companies. This may however also create backlash, as it has done in the past, since residents incur considerable costs. It also needs to be considered how to deal with residents who have opted for individual solutions, such as heat pumps, especially now that natural gas prices render sustainable alternatives more attractive. As of now, there is no coherent policy to keep burdens low and share these equitably. As such, there is no coherent policy package to address the broader repercussions of the heat transition (IP4).

**Organizational multiplicity.** Since the urban heat transition is a large-scale endeavor, many parties are involved in the process. It is however clear that municipalities bear responsibility for driving the transition and the realization of sustainable alternatives. Within their decision-making process, there are opportunities for the participation of residents (see below). Other parties with a clear role in this process are the current heat companies, who are likely to play a role in new DH systems, are distribution system operators of gas and electricity networks, who may be involved in the realization of infrastructure and providers of heat sources, including industry and energy producers. The way each party is involved, and how interests are aligned, depend on the local situation and the role the municipality plays while drafting the heat transition vision. Whether this can bring about tilting the landscape (IP6) will depend on the precise local conditions.

**Inclusivity and local involvement and Ownership and accountability** One of the explicit aims of the neighborhood approach is to include residents and other stakeholders in the decision-making process, from choosing the solutions to execution. This entails both the informal and formal decision-making process. The informal process consists of the neighborhood approach, drafting of the municipal heat transition vision and choice for sustainable heating solutions per neighborhood. Municipalities should organize ample opportunities for participation in this stage, as this concerns the vital choices, but this is legally mandated. The formal process commences when this choice needs to be realized, and where it concerns collective systems such as DH, with the allocation of so-called DH plots and the selection of a heat company. During this stage of the decision-making processes, legally mandated opportunities for participation arise. This participation is key for several reasons, but whether it allows for the destabilizing of the regime (IP3), or to allow to address the broader repercussions (IP4) depends on the local conditions.

Residents can also opt to organize sustainable heat alternatives themselves. Since recent years the number of heat cooperatives have been on the rise; in 2022 110 heat cooperatives were registered [61]. These initiatives aim to create collective systems from bottom-up and through self-organization. Municipalities can create (financial) support instruments for such initiatives. Within the Collective Heating Act, there is a legal basis for these initiatives, but restricts this for initiatives with 500 connections and to small end-users only. It is therefore considered an exemption from the normal situation, in which large-scale systems will be developed by established heat companies (Akerboom & Huygen, 2020). Whether this will allow for heat cooperatives to flourish remains to be seen. As such, this legal exemption does not constitute destabilizing the regime (IP3) and whether it can address the broader repercussions (IP4) of the heat transition remains also to be seen.

**Information, demonstration, and innovation and Experimentation and flexibility.** The Dutch central government has invested in spreading information about the full natural gas phase out. National campaigns have made the public familiar with the goal, and at the local level, interested residents can obtain information about sustainable alternatives from the municipality and energy counters, which can help find the best technological solution and help realize these. The pilot projects, financially supported by the national government, monitor several projects, differing from technological solutions to social innovation, and help distill lessons learned for future projects. There are therefore rather a lot of efforts to monitor progress and learn from past experiences. At the same time, the Dutch Climate Agreement contains agreements on developing standardized solutions, including sustainable alternative and insulation solutions, fit for different housing types. It is unclear whether these agreements have been executed. There is therefore an attempt to stimulate (IP1) and accelerate different niches (IP2).

**In conclusion.** Of the 40 dimensions, 6 have been implemented, 18 have not been implemented, 3 could be improved and of 13 dimensions the full effects need to be studied, see for an overview Table 4. The effectiveness of most policy instruments cannot yet be determined. The heating transition visions have been drafted only in recent years, and given the complexity of the challenge, no significant number of houses have transitioned to an alternative solution. Under the governance of the Dutch Climate Act, there are evaluation methods, such as the Climate and Energy Report (appears annually in October) and a series of meetings set up to monitor the execution of the agreements under the Dutch Climate Agreement. This enables all parties to monitor the progress towards the goals, and when necessary, adjust plans and increase instruments.

**Table 4:** Factors for rapid heating transitions for the Dutch context.

Category	Transition typology/frame	Policy intervention point	Dimension	Present in the Netherlands?
Holistic approach (NEW)∗	De/re-alignment (P2) Transformative change (TF3)	Stimulate different niches (IP1) Accelerate niches (IP2)	1) Options of DHC balanced against alternatives, including environmental and social benefits 2) Availability and relevance of local resources 3) Methodology at the national level 4) Price competitiveness, throughout lifespan 5) Flexible heat and cold production through diversified and complementary optimisation	1) M 2) Y 3) N 4) N 5) N
Adequate national policy and regulatory environment (NEW)	Reproduction (P0) De/re-alignment (P2) Reconfiguration (P4) Transformative change (TF3)	Accelerate niches (IP2) Destabilize the regime (IP3) Coordination multi-regime interaction (IP5)	6) Balance of national and local regulation 7) Connection of heat regulation and urban planning 8) Set of policy tools present 9) Presence of financial incentives: taxes and support schemes 10) Ambitious sustainability targets 11) Study relevance of (temporary) mandatory connections at a local level 12) Transparency of prices 13) Incentives for a competitive energy supply over time	6) N 7) I 8) Y 9) M 10) I 11) Y 12) M 13) N
Equity and co-benefits	Substitution (P3) Reconfiguration (P4)	Destabilize the regime (IP3) Address the broader repercussions (IP4)	14) Use of public funds, loans, subsidies, and taxes 15) Cost-sharing 16) National policy targets 17) Co-benefits and positive externalities	14) M 15) M 16) Y 17) N
Organizational multiplicity	Substitution (P3) Reconfiguration (P4)	Destabilize the regime (IP3) Address the broader repercussions (IP4)	31) Various government actors (local, municipal, provincial, national) 32) Industry and industrial organizations 33) Civil society groups and research institutes 34) Transnational actors 35) Align interests (NEW) 36) Comprehensive project development	31) Y 32) N 33) N 34) N 35) M 36) M
Inclusivity and local involvement	De/re-alignment (P2)	Stimulate different niches (IP1)	18) Emphasis on domestic use 19) Prioritization of rural areas 20) Involvement of users, in relation to prices NEW) 21) Incentives for local private firms and enterprises	18) Y 19) N 20) N 21) N
Ownership and accountability	De/re-alignment (P2) Substitution (P3) Reconfiguration (P4) Transformative change (TF3)	Accelerate niches (IP2) Destabilize the regime (IP3) Address the broader repercussions (IP4)	27) Household or community ownership 28) Improvement of standards 29) Compulsory use requirements and/or penalties for non-compliance 30) Scaling back of subsidies	27) N 28) Y 29) N 30) N
Information, demonstration and innovation	Reproduction (P0) De/re-alignment (P2) Transformative change (TF3)	Stimulate different niches (IP1) Accelerate niches (IP2) Tilt the landscape (IP6)	22) Dissemination of data, information, and knowledge 23) Active marketing or demonstration programs 24) Technological learning 25) Innovations in technical performance 26) Combine technical and nontechnical innovation (NEW)	22) M 23) M 24) M 25) M 26) N
Experimentation and flexibility	De/re-alignment (P2) Transformative change (TF3)	Stimulate different niches (IP1) Accelerate niches (IP2)	37) Experimentation in technical design 38) Multiple uses, applications, or configurations 39) Progression of scalable targets 40) Adaptability in transition management	37) M 38) N 39) N 40) M

Y = yes.

N = no.

I = could be improved.

M = full effects remain to be studied.

## 5 Conclusion and policy implications

In this article we sought to understand how policy studies may contribute to the acceleration of heating transitions. Studies of heating transitions as this point in time focus on the variety of technological solutions, or a specific geographical location but often do not present a policy focus identifying the various technological, financial, and social considerations emerging from a heating transition. By combining policy studies with heating transition literature this article makes one theoretical and one empirical contribution.

First, we aligned transition typologies, consisting of a discussion of transition paths and innovation frames, with policy intervention points. This allows us to understand which type of transition is best stimulated through which intervention point. Second, we provided an overview of existing literature on policy studies of heating transitions, identifying lessons learned and factors necessary for rapid heating transitions. By combining these theoretical frameworks, we identified specifically for the heating transitions which factor constitutes which type of transition and is best stimulated through which intervention point. This provides necessary input and feedback for the (re-)design of (new) policy packages seeking to accelerate and achieve heating transition. This framework may be used in future studies and by policymakers to understand policy gaps and potential failures.

We then applied this framework to the Dutch urban heat transition policy, to test and validate the framework, and identify policy gaps and failures for the Dutch context. Here we highlighted important policy gaps, and identified potential failures for policy effectiveness as often the results of the policy interventions remain unclear. Specifically, two policy intervention points seem important to urban heating transitions, as is also recognised by lessons learned [13,43]: stimulating different niches and addressing the broader repercussions of the transition. Although there is no explicit choice for one technology, there is a focus on collective heating systems, as this is the focus of the first legislative proposal. This way, it seems that decision-making is heavily geared towards realizing collective heating grids, whereas policy instruments stimulating other solutions lack. There is therefore no systematic effort to stimulate different niches. This leads to the policy recommendation to stimulate more actively other options through planning and financial instruments. Moreover, given the exemption status of bottom-up initiatives, and lack of clarity over how financial consequences will be mitigated or prevented, there is no coherent policy addressing the broader repercussions of the heating transitions. This creates uncertainty concerning consequences, and may render households less interested in the transition, despite the potential benefits in terms of climate change mitigation and energy prices. That results in the policy recommendations to increase the financial support for this essential transition.

Literature currently focused on (past) heating transitions do provide valuable insights for policy interventions to overcome the gaps and potential failures. In Denmark for instance, municipalities perform social and costs-benefit analysis to determine various impacts on energy prices, jobs, potential of different energy resources and security of supplies. This instruments thus presents a holistic analysis of impacts and potential of different solutions [62]. Future studies may therefore focus on the potential replicability of these instruments for other contexts but may also focus on new instruments addressing todays social, financial, and technological considerations. Future studies should also empirically how at the neighborhood level these instruments impact the transition. Since most projects are in the development phase, little can yet be concluded about final outcomes. Future studies could however focus on observational methods for ongoing processes.

## Data availability statement

All data used for the analysed in this manuscript, is provided, or referenced to the original source, in the article.

## CRediT authorship contribution statement

**Sanne Akerboom:** Writing – review & editing, Writing – original draft, Methodology, Investigation, Formal analysis, Data curation, Conceptualization.

## Declaration of competing interest

The author declares that they have no known competing financial interests or personal relationships that could have appeared to influence the work reported in this paper.
